# Cardiac Sarcoidosis: Clinical Insights, Diagnosis, and Management Strategies

**DOI:** 10.31083/RCM26605

**Published:** 2025-02-21

**Authors:** Nicola Farina, Giacomo De Luca, Giovanni Peretto, Alex Bartoli, Antonio Esposito, Marco Matucci-Cerinic, Lorenzo Dagna, Corrado Campochiaro

**Affiliations:** ^1^Unit of Immunology, Rheumatology, Allergy and Rare Diseases, San Raffaele Scientific Institute, 20132 Milan, Italy; ^2^Vita-Salute San Raffaele University, 20132 Milan, Italy; ^3^Unit of Arrythmology, San Raffaele Scientific Institute, 20132 Milan, Italy; ^4^Department of Radiology, CHU La Timone, Assistance Publique Hôpitaux de Marseille, 13005 Marseille, France; ^5^Experimental Imaging Center, IRCCS San Raffaele Scientific Institute, 20132 Milan, Italy

**Keywords:** cardiac sarcoidosis, imaging, treatment, biologic

## Abstract

Cardiac sarcoidosis (CS) is a multifaceted inflammatory disease that affects the heart, leading to complications such as arrhythmias, heart failure, and sudden cardiac death. Endomyocardial biopsy is the diagnostic gold standard, but its low sensitivity and risks limit its utility. Imaging modalities, such as cardiac magnetic resonance and positron emission tomography, are critical for diagnosing and managing CS. Additionally, CS treatment primarily involves corticosteroids and immunosuppressive agents to reduce inflammation and control disease progression, although biologics such as tumor necrosis factor-alpha (TNF-α) inhibitors are considered in refractory or steroid-dependent cases. This narrative review revises the existing knowledge on the diagnosis and treatment of CS, providing a comprehensive overview of current strategies.

## 1. Introduction

Sarcoidosis is a multisystem inflammatory disorder characterized by the formation of non-caseating granulomas in various organs, most commonly affecting the lungs and intrathoracic lymph nodes [1]. Despite being a relatively rare condition, the diverse clinical manifestations and unpredictable course of sarcoidosis pose significant challenges for its diagnosis and management [2]. While pulmonary involvement is the hallmark of sarcoidosis, its impact on other organs, including the heart, has gained recognition for its clinical significance and potential complications [3].

Cardiac sarcoidosis (CS) represents a unique and potentially life-threatening manifestation of sarcoidosis involving the heart. Although precise epidemiological data remain elusive, CS is increasingly recognized as a significant cause of morbidity and mortality among sarcoidosis patients [3]. The clinical presentation of CS varies widely, ranging from asymptomatic electrocardiographic abnormalities to fatal arrhythmias, heart failure, and sudden cardiac death [4]. The insidious nature of CS, coupled with its heterogeneous clinical features and nonspecific symptoms, often leads to diagnostic delays or misdiagnosis, emphasizing the need for heightened awareness and improved diagnostic strategies [5].

Understanding the pathophysiology of CS is crucial for elucidating its clinical manifestations and guiding therapeutic interventions. The inflammatory cascade underlying sarcoidosis can affect any heart layer, disrupting the electrical conduction system, impairing contractility, and promoting arrhythmogenesis [6]. Moreover, the unpredictable nature of granuloma formation and regression in the myocardium further complicates the clinical course of CS, necessitating a multidisciplinary approach for optimal management [6].

Recently, advances in imaging modalities, such as cardiac magnetic resonance imaging (MRI) and positron emission tomography (PET), have revolutionized the diagnosis and monitoring of CS, enabling early detection of cardiac involvement and guiding therapeutic decisions. Despite these advancements, challenges persist in accurately diagnosing CS, particularly in differentiating it from other cardiac conditions with overlapping clinical features [6].

This review aims to provide a comprehensive overview of the epidemiology, pathophysiology, clinical manifestations, diagnostic approaches, and management strategies for cardiac sarcoidosis. By synthesizing the latest evidence and clinical insights, we seek to enhance awareness and promote optimal care for patients with this complex, often under-recognized condition.

This narrative review revises the existing knowledge on the diagnosis and treatment of CS, aiming to provide a comprehensive overview of current strategies for identifying, monitoring, and managing this complex condition. By consolidating the latest evidence and expert recommendations, it seeks to guide clinicians in optimizing diagnostic accuracy, improving imaging techniques, and tailoring therapeutic approaches to enhance patient outcomes.

## 2. Etiology and Pathogenesis

Despite extensive research, the exact etiology of sarcoidosis still needs to be discovered; however, sarcoidosis is widely believed to result from an interplay of genetic predisposition, environmental triggers, and dysregulated immune responses [1]. Several studies have identified associations with specific human leukocyte antigen (HLA) alleles, particularly HLA-DRB1 and HLA-DQB1, indicating a genetic predisposition to sarcoidosis development [7, 8, 9]. Additionally, genome-wide association studies have highlighted several candidate genes involved in immune regulation and inflammation, further supporting the genetic component of sarcoidosis [10].

Environmental factors are thought to trigger the development of sarcoidosis in genetically susceptible individuals [1]. Various environmental exposures, including infectious agents (such as mycobacteria, Cutibacterium acnes, and viruses), occupational hazards (such as beryllium, silica, and organic dust), and unidentified antigens, have been implicated as potential triggers [11]. However, no single causative agent has been consistently identified across all cases, suggesting a complex interplay between multiple environmental factors.

The exaggerated immune response that characterizes sarcoidosis pathogenesis is driven by the activation of T lymphocytes and the formation of granulomas [11]. Antigen-presenting cells, such as macrophages and dendritic cells, process and present environmental or self-antigens to CD4+ T helper cells, leading to the release of proinflammatory cytokines, including tumor necrosis factor-alpha (TNF-α), interleukin-2 (IL-2), and interferon-gamma (IFN-γ) [12]. These cytokines drive the recruitment and activation of additional immune cells, perpetuating the inflammatory cascade and granuloma formation [11].

The pathogenesis of CS shares similarities with systemic sarcoidosis; however, the unique microenvironment of the myocardium and the presence of specialized cardiac cells contribute to distinct clinical manifestations and complications. In CS, inflammatory infiltrates comprising activated macrophages, T lymphocytes, and multinucleated giant cells infiltrate the myocardium, forming non-caseating granulomas [13]. Similar to systemic sarcoidosis, the exact mechanisms triggering myocardial inflammation in CS remain incompletely understood but likely also involve a combination of genetic predisposition, environmental triggers, and dysregulated immune responses [1].

Granulomas within the myocardium can disrupt the normal architecture and function of the heart, leading to fibrosis, myocardial scarring, and conduction abnormalities. Cytokine-mediated inflammation and oxidative stress can further impair cardiomyocyte function, resulting in myocardial dysfunction and heart failure [13]. CS predisposes individuals to various arrhythmias and conduction abnormalities, including atrioventricular block, ventricular tachycardia, and sudden cardiac death, due to the disruption of the cardiac conduction system by granulomas or inflammation [14].

## 3. Epidemiology

Cardiac involvement occurs in approximately 5% of patients with sarcoidosis, although autopsy studies have suggested that the prevalence might be higher, ranging from 20% to 30% [13]. CS typically affects individuals between the ages of 20 and 60 years, with incidences peaking between 40 and 50 years of age. Moreover, men appear to have a slightly higher prevalence than women [15]. Notably, the prevalence of CS varies across different populations and geographic regions, whereby studies have reported higher rates of CS in Japan compared to Western countries, likely due to differences in genetic predisposition and environmental exposures [16]. The majority of patients with CS also have pulmonary involvement, with estimates ranging from 50% to 90%. Therefore, pulmonary sarcoidosis is considered a significant risk factor for cardiac involvement, particularly in patients with extensive pulmonary fibrosis or respiratory impairment [1].

## 4. Clinical Manifestations

Sarcoidosis can affect any part of the heart, including the myocardium, endocardium, pericardium, and the conduction system. Cardiac symptoms vary depending on the location, extent, and activity of the disease [17]. The most commonly observed manifestations are cardiac arrhythmias, often presenting as syncope or palpitations [18]. Conduction abnormalities, such as advanced atrioventricular (AV) block, are reported in up to 42% of CS cases [19], typically due to the septal localization of granulomas infiltrating the conduction system [20]. Although transient recovery of AV conduction may occur either spontaneously or with immunosuppressive therapy [21], permanent pacing is often required due to the unpredictable course of the disease [22].

Among tachyarrhythmias, ventricular arrhythmias are the most common at presentation. These include frequent ectopic beats, unsustained ventricular tachycardia, and more severe arrhythmias, such as sustained ventricular tachycardia or ventricular fibrillation [17, 18]. Life-threatening ventricular arrhythmias, sudden cardiac death, and cardiocirculatory arrest account for approximately 30% of CS clinical manifestations [22]. Even in patients with bradyarrhythmia, the mid- to long-term risk of malignant ventricular arrhythmias is sufficiently high that guidelines recommend the implantation of a cardioverter defibrillator rather than a pacemaker [23, 24]. Many patients present with several morphologies of ventricular tachycardias or electrical storms due to the presence of an extensive and complex arrhythmic substrate [25, 26]. In acute sarcoidosis, catheter ablation has poor outcomes [25, 26] and should be postponed until the post-inflammatory stage, as identified by multimodality imaging [27].

Granulomatous infiltration of the atria, causing local inflammation and scarring, may also be responsible for supraventricular tachyarrhythmias, particularly atrial fibrillation and focal atrial tachycardias [19, 28]. Catheter ablation has poor outcomes even in atrial disease, and immunosuppression may help reduce the arrhythmic burden [23]. Heart failure is present in approximately 17% of patients with manifested CS [17]. Meanwhile, presentation with acute decompensated heart failure often results from life-threatening arrhythmias [22]. As recommended by international guidelines, hemodynamically unstable patients may require transfer to tertiary care centers for mechanical circulatory support [23]. Sarcoidosis can also lead directly to ventricular systolic dysfunction, spanning the spectrum from dilated to non-dilated left ventricular cardiomyopathies [29]. Most patients have mildly to moderately reduced left ventricular ejection fraction (LVEF) and signs of diastolic dysfunction [17]. Meanwhile, for stable patients with dyspnea, differential diagnosis should consider pulmonary disease. Right ventricular involvement, while rare in other forms of myocarditis [30], is a common sign of mechanical dysfunction in CS and necessitates differentiation from arrhythmogenic cardiomyopathy [31]. When significant interventricular conduction delays occur, such as from a complete left bundle branch block, cardiac resynchronization therapy is the preferred treatment option [32].

**Fig. 1.**
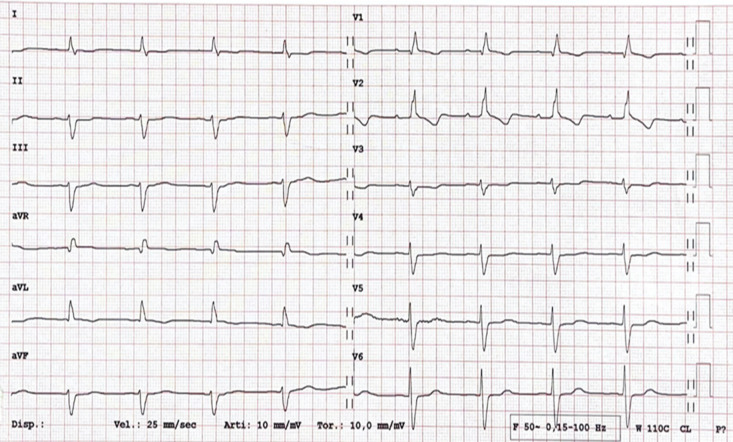
**A 12-lead electrocardiogram (ECG) in a 46-year-old male with a recent history of syncope**. A first-degree atrioventricular block, complete right bundle branch block, and left anterior fascicular block are shown (trifascicular block). Diagnosis of cardiac sarcoidosis with involvement of the interventricular septum was confirmed by both histology and multimodality imaging. The patient underwent dual-chamber transvenous cardioverter defibrillator implantation. aVR, augmented vector right; aVL, augmented vector left; aVF, augmented vector foot.

In subclinical CS, the 12-lead electrocardiogram (ECG) is often the first exam to reveal abnormalities during screening, even without symptoms [17]. As illustrated in Fig. 1, the presence of a trifascicular block or unexplained conduction system disease in young or middle-aged patients warrants consideration of CS, among other diseases that affect the interventricular septum [33].

## 5. Diagnosis

Considering the diverse clinical manifestations of sarcoidosis and the lack of a single definitive diagnostic test, a diagnosis of sarcoidosis entails a multifaceted approach [34]. The initial assessment often involves a thorough clinical evaluation to identify symptoms such as a cough, dyspnea, fatigue, or skin lesions, which may prompt further investigation [35]. Imaging studies, including chest X-rays and high-resolution computed tomography scans, are instrumental in detecting pulmonary involvement and assessing disease extent [36]. Laboratory tests, such as analyzing serum angiotensin-converting enzyme levels, can provide supportive evidence, although these lack specificity [37]. A definitive diagnosis typically requires histological confirmation of non-caseating granulomas obtained through a biopsy of affected tissues, such as the lungs, skin, or lymph nodes [38].

Diagnosing CS presents unique challenges due to its variable clinical presentation and potential for life-threatening complications [39]. Distinct from more frequent presentations of sarcoidosis, CS requires specific cardiac-focused tests. Initial screening often involves an ECG alongside echocardiography to assess for conduction abnormalities, arrhythmias, or structural changes. However, these tests may lack sensitivity for early-stage disease occurrences [40]. Advanced imaging techniques, such as cardiac magnetic resonance (CMR) imaging and PET, are valuable for detecting myocardial inflammation and assessing disease severity [41]. Endomyocardial biopsy (EMB) remains the gold standard for confirming CS but is invasive and carries risks [42]. Given the challenges in diagnosing CS, a high index of suspicion, multidisciplinary collaboration is essential between cardiologists, pulmonologists, and rheumatologists, and the utilization of complementary diagnostic modalities are essentialto ensure the accurate diagnosis and timely initiation of treatment.

### 5.1 Cardiac Imaging

While EMB remains the gold standard for diagnosing CS, it has low sensitivity and presents a serious complication rate of around 1% [43, 44]. Thus, a combination of extracardiac histological confirmation and myocardial involvement in imaging is sufficient for diagnosing CS [14]. Despite its low sensitivity, trans-thoracic echocardiography (TTE) is often the first-line imaging tool [45]. Findings may include strain abnormalities and changes in myocardial thickness, although the exam can sometimes appear normal; meanwhile, the disease progression often leads to diastolic dysfunction, with LVEF decreasing in advanced stages [46].

**Fig. 2.**
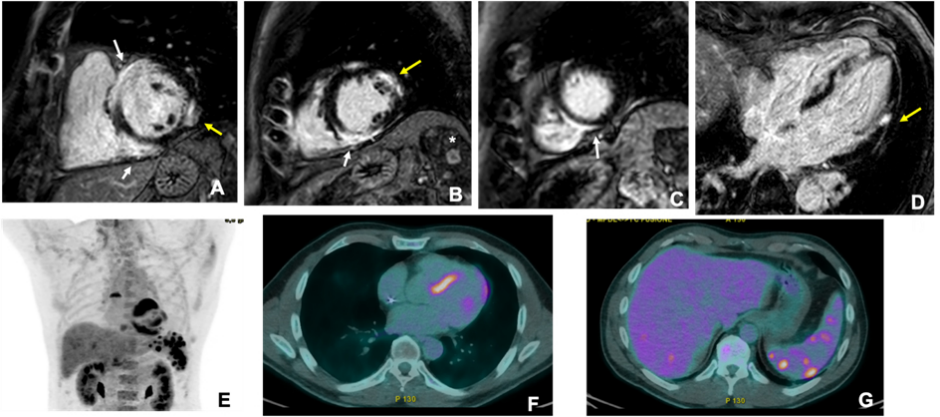
**Cardiac magnetic resonance (CMR) and 18F-fludeoxyglucose (FDG)-positron emission tomography (PET) images of a male adult (51 years old) 24 hours after resuscitation from sudden cardiac death**. CMR (upper panels) showed hypertrophy of the mid-basal interventricular septum, associated with patchy areas of late gadolinium enhancement (LGE) with a subepicardial pattern involving the entire septum, especially at the junction point with the right ventricle, «hook sign», (white arrows in (A–C)). Smaller areas of subepicardial (LGE) were also evident on the lateral wall (yellow arrows in (A,B,D)). Additionally, magnetic resonance imaging (MRI) showed multiple enhancing nodules in the spleen (asterisk in (B)), suggestive of granulomas. The patient also underwent 18F-FDG-PET (lower panels), which showed marked uptake in the site of LGE (E,F) and focal areas of uptake on the liver and the spleen (G).

CMR and PET are the primary imaging modalities for CS and can be used to assess myocardial changes and disease activity. CMR provides comprehensive information on cardiac anatomy, function, and myocardial composition, including edema, fibrosis, and scarring [47]. However, CS can mimic various cardiomyopathies, with nonspecific findings such as localized dyskinesia, septo-basal thinning, or pericardial effusion [48]. T2-weighted imaging detects myocardial edema, typically in the septum [49]. Thus, late gadolinium enhancement (LGE) is crucial for diagnosing CS, revealing fibrosis in patchy, multifocal patterns sparing the endocardium [50]. As shown in Fig. 2, LGE is often found in the basal and mid-septum, with potential extension to the right ventricle [51], and it is a key prognostic factor [17]. Studies have shown a significant association between LGE and adverse outcomes, including ventricular arrhythmias and mortality [52, 53].

Nuclear imaging, particularly cardiac 18F-fludeoxyglucose (FDG)-PET, plays a central role in detecting myocardial inflammation, with high sensitivity (94–100%) [54]. 18F-FDG-PET is more sensitive than Gallium scintigraphy, Thallium, or Technetium-99m single-photon emission computed tomography for detecting myocardial inflammation [55]. To suppress normal myocardial glucose uptake, patients undergo a specific high-fat, low-carbohydrate diet followed by fasting, which can sometimes lead to inconclusive results in 10–15% of cases [56, 57]. PET scans often show patchy or diffuse myocardial uptake, particularly in the basal septum (Fig. 2). A combined perfusion scan enhances specificity, revealing perfusion defects associated with fibrosis or microcirculation abnormalities [58]. Both “mismatch patterns” of FDG uptake and right ventricular involvement can predict adverse cardiovascular outcomes [59, 60]. PET is also used to monitor treatment responses to immunosuppression [61]. Hybrid PET/MRI scanners combine LGE and FDG uptake and offer a deeper characterization of CS and differentiation between active and inactive disease [62]. Thus, using hybrid PET/MRI scanners also allows for better prognostication, with a lower radiation dose and higher diagnostic performance than individual PET/computed tomography (CT) [63, 64].

Although MRI is the preferred non-invasive tool for myocardial characterization, CT with late contrast enhancement has also shown good accuracy in identifying myocardial scars in inflammatory cardiac diseases [65]. Thus, CT may be particularly useful in patients with implanted devices and for initial assessments in cases where lung involvement is more prevalent [66]. Additionally, combining CT with 18F-FDG-PET can simultaneously provide information about myocardial scarring and disease activity.

### 5.2 Endomyocardial Biopsy

As reported above, while EMB is the gold standard for the definitive diagnosis of CS as it provides histopathological confirmation of non-caseating granulomas in the myocardium [42], its use in clinical practice is limited by several factors [67]. The primary challenge is the often patchy distribution of granulomas in CS, which can lead to a low diagnostic yield. Studies have shown that the sensitivity of EMB in detecting granulomas in CS can range from 20% to 30%, largely because standard biopsy techniques sample small areas of the heart, often missing the regions involved in the disease. In addition, EMB carries risks, such as cardiac perforation, arrhythmias, and damage to the tricuspid valve, which further limits its widespread use in diagnosing CS [42].

Advancements in imaging technology have enhanced the utility of EMB by improving the ability to target affected areas of the myocardium. CMR and PET allow for identifying areas of active inflammation and scarring. These imaging modalities can guide biopsies to areas of abnormal tissue, significantly increasing the likelihood of detecting granulomas and improving the diagnostic yield of the procedure. In cases where EMB is performed using these targeted approaches, sensitivity may increase, making the biopsy a more valuable diagnostic tool [41].

Despite its limitations, EMB remains an important part of the diagnostic algorithm for CS, particularly in cases where other diagnostic tools fail to provide conclusive evidence. For example, it is especially useful in differentiating CS from other forms of myocarditis, such as giant cell myocarditis, which shares certain clinical and imaging characteristics with CS but has a more aggressive prognosis and requires different treatment strategies. Therefore, a histopathological diagnosis from EMB can significantly impact clinical management decisions, particularly in guiding immunosuppressive therapy or ruling out alternative diagnoses.

The decision to perform an EMB must be carefully considered and based on a balance of clinical suspicion, non-invasive imaging findings, and the potential impact of the biopsy on patient management. Given the risks associated with the procedure, EMB is generally reserved for cases where the diagnosis remains uncertain after extensive evaluation through less invasive methods. In these cases, the diagnostic value of confirming CS through biopsy may outweigh the procedural risks, particularly in patients where establishing the diagnosis would lead to more aggressive treatment strategies. Therefore, while not routinely performed in all suspected cases of CS, EMB can play a decisive role in confirming the diagnosis in selected cases where histological confirmation is essential [42]. Table 1 summarizes the main strengths and weaknesses of PET, CMR, and EMB.

**Table 1.** **Comparison of main diagnostic modalities used in the suspect of cardiac sarcoidosis**.

Modality	Strengths	Weaknesses	Best-use cases
Endomyocardial Biopsy	Gold standard with definitive histopathological confirmation of non-caseating granulomas. Improved yield with imaging-guided biopsy.	Low sensitivity (20–30%) due to patchy granuloma distribution. Risk of complications (1%), such as perforation and arrhythmias.	Differentiating CS from other myocarditis (e.g., giant cell myocarditis). Cases where histological confirmation impacts treatment.
Cardiac Magnetic Resonance	Non-invasive and comprehensive. Detects edema (T2-weighted imaging), fibrosis, and scarring (LGE). Strong prognostic value (e.g., LGE and mortality).	Requires gadolinium, limiting use in severe renal dysfunction. Less effective for assessing active inflammation compared to PET.	Preferred tool for myocardial characterization and prognosis. Guiding endomyocardial Biopsy to affected areas.
18F-FDG PET	High sensitivity (94–100%) for detecting inflammation. Useful for monitoring treatment response. Combined PET/CT improves specificity.	Requires meticulous patient preparation (e.g., dietary protocol). 10–15% of scans may be inconclusive due to physiological uptake variability.	Evaluating active inflammation. Monitoring response to immunosuppressive therapy.

CS, cardiac sarcoidosis; CT, computed tomography.

## 6. Treatment

Sarcoidosis treatment aims to alleviate symptoms, prevent organ damage, and improve quality of life [68, 69, 70, 71]. However, management strategies can vary depending on disease severity, organ involvement, and individual patient factors. Therapeutic interventions encompass pharmacological agents, immunomodulatory therapy, and supportive measures tailored to specific organ manifestations [68].

Corticosteroids are the mainstay of pharmacological therapy for both extracardiac and cardiac manifestations of sarcoidosis, exerting anti-inflammatory effects to suppress granuloma formation and reduce disease activity [72]. Given the potentially life-threatening nature of CS and its association with poor prognosis, higher doses of corticosteroids may be required to control cardiac inflammation. Intravenous pulse methylprednisolone therapy or high-dose oral prednisone regimens may also be utilized to induce remission in severe cases of CS [73].

In addition to corticosteroids, immunosuppressive agents such as methotrexate or azathioprine may be considered steroid-sparing agents or adjunctive therapy in refractory cases. Meanwhile, biologic agents, such as tumor necrosis factor-alpha (TNF-α) inhibitors (e.g., infliximab, adalimumab) and JAK inhibitors, have demonstrated efficacy in refractory cases or steroid-dependent disease [74, 75]. Anti-TNF agents may be particularly beneficial in patients with extrapulmonary manifestations, such as CS, neurosarcoidosis, or ocular involvement [12].

## 7. American Heart Association Recommendations

The latest American Heart Association (AHA) guidelines, published in 2024, provide a detailed framework for diagnosing and managing CS and emphasize the multidisciplinary and evidence-informed approach previously highlighted throughout this review [76]. These guidelines highlight the complexity of CS diagnosis, given its variable clinical presentations and the challenges in confirming myocardial involvement. The AHA also outlines a tiered treatment strategy, starting with corticosteroids and advancing to immunosuppressive and biological therapies based on disease severity and response to treatment.

### 7.1 Diagnosis

According to the AHA guidelines and in line with the previously summarized data above, diagnosing CS requires a combination of clinical assessment, advanced imaging modalities, and, when possible, histological confirmation. CMR and PET are the cornerstone imaging techniques, providing critical information about inflammation and myocardial scarring. While CMR offers high sensitivity and prognostic insights, PET detects active granulomatous inflammation and guides therapeutic monitoring. Thus, EMB remains the gold standard but is limited by a low diagnostic yield due to the patchy distribution of granulomas.

### 7.2 Treatment

The AHA guidelines advocate for a stepwise approach to CS management. Corticosteroids are the first-line therapy, with prednisone (30–40 mg/day) being the standard initial treatment. For severe or life-threatening cases, such as ventricular arrhythmias or cardiogenic shock, high-dose intravenous methylprednisolone may be used to control inflammation rapidly. In cases of steroid-resistant or relapsing disease, steroid-sparing agents such as methotrexate, azathioprine, or mycophenolate are recommended. Biologic therapies, including TNF-α inhibitors such as infliximab and adalimumab, are reserved for refractory cases or situations requiring aggressive intervention early in the disease course. Meanwhile, treatment is tailored to each patient through regular follow-up and imaging-based monitoring, aiming to control inflammation, prevent progression, and improve outcomes. The guidelines underscore the importance of individualized care and interdisciplinary collaboration in managing this challenging condition.

## 8. Prognosis

The prognosis of CS varies depending on several factors, including the extent of myocardial involvement, clinical presentation, and certainty of diagnosis. Recent cohort studies have reported encouraging survival rates, with 5-year survival consistently at 90% or higher. Poor outcomes are associated with sustained ventricular tachycardia or heart failure at presentation, while atrioventricular block alone tends to promote a better prognosis. Comparatively, *de novo* or clinically isolated presentations predict worse outcomes, often due to delayed diagnoses and more advanced disease stages. Interestingly, a histologically confirmed diagnosis of myocardial CS portends a worse prognosis compared to a probable diagnosis based on extracardiac findings [14].

Although these factors offer insights into prognosis, it remains unclear whether current treatments significantly improve long-term outcomes. Therefore, further studies are needed to assess the impact of therapeutic interventions on survival and quality of life in CS patients.

## 9. Conclusions

Sarcoidosis and CS are complex inflammatory disorders that require a multifaceted management approach. While pharmacological agents target systemic inflammation and immune dysregulation, the management of cardiac sarcoidosis necessitates a dual focus on immunomodulatory therapy and cardiological interventions to optimize outcomes and prevent cardiac complications. Multidisciplinary collaboration between rheumatologists, pulmonologists, cardiologists, and other specialists is essential for individualized treatment planning and comprehensive care of patients with sarcoidosis.
